# Construction of homologous cancer cell membrane camouflage in a nano-drug delivery system for the treatment of lymphoma

**DOI:** 10.1186/s12951-020-00738-8

**Published:** 2021-01-06

**Authors:** Qiangqiang Zhao, Xiaoying Sun, Bin Wu, Yinghui Shang, Xueyuan Huang, Hang Dong, Haiting Liu, Wansong Chen, Rong Gui, Jian Li

**Affiliations:** 1 Department of Blood Transfusion, The Third Xiangya Hospital, Central South University, Changsha, 410013 People’s Republic of China; 2 Department of Hematology, The Qinghai Provincial People’s Hospital, Xining, 810007 People’s Republic of China; 3 School of Nursing, Medical College, Soochow University, Suzhou, 215006 People’s Republic of China; 4 Department of Emergency, The Qinghai Provincial People’s Hospital, Xining, 810007 People’s Republic of China; 5 Department of Transfusion Medicine, Tongji Medical College, Wuhan Hospital of Traditional Chinese and Western Medicine, Huazhong University of Science and Technology, Wuhan, 430022 People’s Republic of China; 6 College of Chemistry and Chemical Engineering, Central South University, Changsha, 410083 People’s Republic of China

**Keywords:** Cancer cell membrane, Mesoporous silica nanoparticles, Isoimperatorin, Apoptosis, Lymphom

## Abstract

**Background:**

Non-Hodgkin’s lymphoma (NHL) possesses great heterogeneity in cytogenetics, immunophenotype and clinical features, and chemotherapy currently serves as the main treatment modality. Although employing monoclonal antibody targeted drugs has significantly improved its overall efficacy, various patients continue to suffer from drug resistance or recurrence. Chinese medicine has long been used in the treatment of malignant tumors. Therefore, we constructed a low pH value sensitivity drug delivery system based on the cancer cell membrane modified mesoporous silica nanoparticles loaded with traditional Chinese medicine, which can reduce systemic toxicity and improve the therapeutic effect for the targeted drug delivery of tumor cells.

**Results:**

Accordingly, this study put forward the construction of a nano-platform based on mesoporous silica nanoparticles (MSNs) loaded with the traditional Chinese medicine isoimperatorin (ISOIM), which was camouflaged by the cancer cell membrane (CCM) called CCM@MSNs-ISOIM. The proposed nano-platform has characteristics of immune escape, anti-phagocytosis, high drug loading rate, low pH value sensitivity, good biocompatibility and active targeting of the tumor site, blocking the lymphoma cell cycle and promoting mitochondrial-mediated apoptosis.

**Conclusions:**

Furthermore, this study provides a theoretical basis in finding novel clinical treatments for lymphoma.

## Background

Non-Hodgkin’s lymphoma (NHL) is a malignant disease of the hematological system, which is commonly encountered globally. At present, the incidence of NHL demonstrates a significant upward trend in different stages of age [1]. Certain patients with NHL can achieve complete remission after a short duration of chemotherapy under the R-CHOP regimen [2]. Unfortunately, about 50% of such patients will relapse, and the 3-year overall survival rate after recurrence is about 30% [3]. Patients with recurrent cancer/NHL have serious side effects due to the use of high-dose chemotherapy drugs, and recurrent lymphoma is insensitive to follow-up treatment using the initial chemotherapy regimen and displays cross-resistance for a variety of anticancer drugs [4]. Therefore, identifying novel anticancer drugs with high efficiency and low toxicity in the treatment of NHL is a vital topic in research.

Recently, natural Chinese herbal medicine has shown to be a valuable source for the screening of anticancer drugs [5]. Isoimperatorin (ISOIM) belongs to furan coumarins, which mainly distributes in Umbelliferae and Rutaceae including Angelica dahurica, Radix Notopterygii, Fructus Cnidii and Radix Glehniae [6]. Studies have illustrated that ISOIM possesses anti-tumor properties. In this regard, Kim YK et al. found that ISOIM has different inhibitory effects on human lung cancer cell line A549 as well as human ovarian cancer cell line SK-OV-3 in a dose-dependent manner [7]. In addition, studies have shown that ISOIM can inhibit the proliferation and promote apoptosis of human gastric cancer cells [8, 9]. However, most anticancer drugs are low molecular weight compounds that are easily excreted via glomerular filtration or liver metabolism [10].

Numerous investigations have shown that nano-carriers loaded with active components of traditional Chinese medicine (TCM) prevent damage caused by light, pH and enzymes, thus maintaining its stability and improving its solubility and bioavailability of drugs [11–13]. Ivanisevic et al. has proved that mesoporous silica nanoparticles (MSNs) has garnered increasing attention due to its good biocompatibility, large specific surface area, and adjustable pore size [14]. Juère et al [15] encapsulated resveratrol in MSNs and found that encapsulated resveratrol greatly improved its physical and chemical properties as well as its biological activity. MSNs may be delivered to tumor cells in triple negative breast cancer by loading chemotherapeutic drugs and siRNA to kill the cancerous cells. Although employing unmodified MSNs as a carrier for drug delivery may improve the biological stability of antineoplastic drugs as well as the permeability of malignant tumor cells, tumor targeting remains insufficient, limiting the application of MSNs in tumors  [16].

Inspired by the near-perfect structure and function of biology in nature, bionic technology has been widely researched [17, 18]. Erythrocyte membranes [19], leukocyte membranes [20] and platelet membranes [21] are all used to construct nano-drug delivery systems. At the same time, Ca^2+^-dependent proteins are often highly expressed on cancer cell membranes (CCM), which mediate the adhesion and targeting of tumor cells [22–24]. Specifically, such properties stimulate tumor cells to recognize and adhere to each other and resist apnesia and apoptosis in vivo [25,26]. Therefore, CCM as a biomimetic nano-system shows strong tumor targeting potential.

**Fig. 1:**
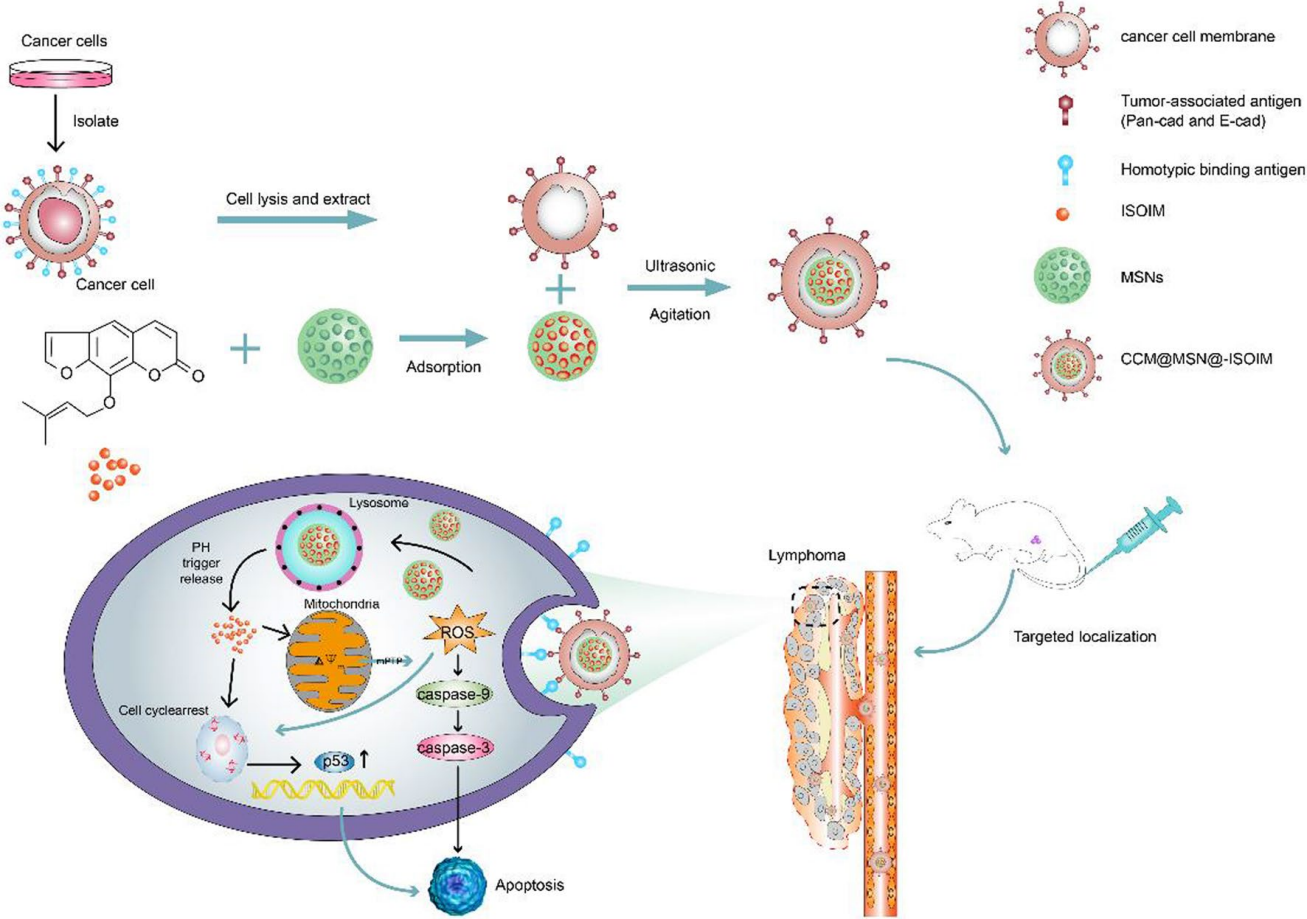
Schematic diagram of CCM@MSNs-ISOIM construction and its targeted therapeutic mechanisms in lymphoma

In this study, MSNs-loaded ISOIM was designed and constructed as the nanoparticles’ core (MSNs-ISOIM), which was encapsulated within the membrane of lymphoma cells, in order to construct a novel nano-targeted drug delivery system (CCM@MSNs-ISOIM) and analyze its anti-tumor outcomes (Fig. 1). We believe that CCM@MSNs-ISOIM has introduced a new direction for the functionalization of inorganic nanomaterials and will accelerate the clinical transformation of nano-drugs.

## Materials and methods

### Materials

Mesoporous silica dispersion was purchased from nanoComposix Company (USA), and Isoimperatorin from DESITE (China). Fetal bovine serum and RPMI1640 cell culture medium were extracted by Gibco (USA). Cell counting kit-8 (CCK-8) was purchased from Dojindo laboratories (Japan), and the Annexin V-FITC/PI apoptosis detection reagent was produced by BD (USA). Cell cycle detection kit, reactive oxygen species (ROS) detection kit, and DAPI were acquired from Beyotime Biotechnology (China). Rhodamine 123 (Rh123) was provided by Yeasen Biotechnology (China). Cy5 and membrane (2 kD) were purchased from Solarbio (China). Cell lysate, Protease inhibitor and TUNEL reaction solution were obtained from Boster (China). Anti-Ki-67, anti-P53, anti-Caspase-9 and anti-Caspase-3 antibodies were all purchased from Servicebio (China), while anti-Pan-Cadherin and anti-E-Cadherin antibodies were acquired from CST (USA). H&E staining kit was purchased from keygen BioTECH (Chnia), and human lymphoma cell line OCI-LY10 was acquired from the Shanghai Institute of Cell Research, Chinese Academy of Sciences.

### Cell culture

OCI-LY10 cells were inoculated in RPMI160 medium containing 10% fetal bovine serum and placed in an incubator at 37 ℃ and 5% CO_2_, which was passed on every 2–3 days. The logarithmic growth phase cells were selected for the experiment.

### Synthesis of MSNs@ISOIM

MSNs (10 mg) and ISOIM (8.9 mg) were added to 5 mL anhydrous alcohol and stirred for 6 h at room temperature, after which free ISOIM was removed via 2 kD dialysis membrane. The samples after dialysis were used to detect the concentration of ISOIM [27]. Entrapment efficiency (EE%) = (Total ISOIM-Total ISOIM in the supernatant)/Total ISOIM × 100%. Loading efficiency (LE%) = (Total ISOIM-Total ISOIM in supernatant)/[(Total ISOIM-Total ISOIM in supernatant) + Total MSNs] × 100%.

### Cancer cell membrane (CCM) extraction

OCI-LY10 tumor cells were dispersed in 5 mL 10% sucrose hypotonic solution containing protease inhibitors and placed on ice for 1 h. The cells were then stirred and homogenized with a high-speed disperser (12,000 rpm × 5 min). After the homogenate was centrifuged at 3000 rpm × 5 min, the supernatant was discarded, and the procedure was repeated 5 times for the remaining precipitation. All cell lysates were collected and centrifuged using a sucrose solution (50% wt, 40% wt and 30% wt) for ultracentrifugation (12,000 rpm × 5 min). The interface components of 30–40% were collected, and the precipitates were collected after 12,000 rpm centrifugation for 10 min. Afterward, the precipitates were washed with ddH_2_O 3 times to obtain the extracted cancer cell membrane (CCM) [28].

### Construction of CCM@MSNs-ISOIM

CCM was mixed with the same volume of MSNs@ISOIM and fused using ultrasound (3 min 42 kHz, 100W), which was filtered 30 times using a filter with a pore diameter of 200 nm. After collecting the mixture and centrifuging at (2000 rpm × 5 min), the excess CCM was removed, and the final CCM@MSNs-ISOIM was obtained.

### Characterization of CCM@MSNs-ISOIM

The liquid transfer gun dripped 10 µL CCM@MSNs-ISOIM onto the copper mesh covered with a supporting film, and the absorbent paper absorbed the excess water. After drying naturally, the morphology of the nanoparticles was observed under a transmission electron microscope (Tecnai G2 Spirit TEM, FEI, USA). The particle size and surface charge of the nanoparticles were determined by Zetasizer Nano ZS (Malvern Nano series, Malvern, UK). The test was carried out at room temperature with 1 mg/mL sample of 1.5 mL. The CCM protein and CCM@MSNs protein were detected by sodium dodecyl sulfate-polyacrylamide gel electrophoresis (SDS-PAGE) to identify whether the MSNs were successfully encapsulated in CCM. UV/Vis spectroscopy (ScanDrop, Analytik Jena, Germany) was used to detect the absorbance of CCM, MSNs, ISOIM and CCM@MSNs-ISOIM, respectively.

### CCM@MSNs-ISOIM release properties for ISOIM

The drug release test of CCM@MSNs-ISOIM was carried out at pH 7.4 and pH 5.0 to determine whether ISOIM could be released according to low pH value sensitivity. Dialysis was applied to analyze the release of ISOIM from MSNs@ISOIM and CCM@MSNs-ISOIM. Dialysis bags (molecular weight cut-off, MWCO 8000–14,000 Da) were filled with 2 mL of MSNs@ISOIM and CCM@MSNs-ISOIM, respectively, while the bags were immersed in 20 mL of PBS containing 0.2% (w/v) Tween 80 with stirring at100 rpm at 37 °C [29, 30]. At fixed times, a sample of release medium (100 µL) was extracted and the same volume of fresh medium was added back to keep the final volume constant. The dialysate was collected and the absorbance of ISOIM was determined at the wavelength of 230.5 nm. A standard curve was established to calculate the cumulative release of ISOIM.

### Biocompatibility of CCM@ MSNs-ISOIM

Five different concentrations of MSNs were added to the suspended red blood cells along with five different concentrations of CCM@MSNs-ISOIM, all of which served as the experimental group. Ultrapure water was used as the positive control with PBS as the negative control. The experimental, positive control and negative control groups were incubated at 37 ℃ for 2 h at 1000 rpm centrifugation for 5 min to collect the supernatant. The supernatant was then added to a 96-well plate. The absorbance (OD) of the supernatant at 540 nm was measured using a multi-function enzyme labeling instrument (PerkinElmer EnSpire, USA). OCI-LY10 cells were inoculated in a 24 well plate (1 × 10^5^ cells/well) and incubated at different concentrations of MSNs and CCM@MSNs (10, 20, 40, 80 and 100 µg/mL, the concentration of MSNs in the above-mentioned two groups was identical). After 24 h of incubation, 10 µL Cell Counting Kit-8 (CCK-8) solution was added to each hole and incubated for 4 h. The absorbance of the solution was determined to be 450 nm. The hemolysis rate (%) = (ODs-ODn)/(ODp-ODn) × 100%, (ODs, ODn and ODp were the absorbance values of the supernatant of the experimental group, negative control and positive control, respectively). The experiment was repeated three times. In order to detect the immune escape ability of CCM@MSNs, RAW264.7 cells were inoculated in a 6-well plate (2 × 10^5^cells/well) and incubated with CCM@MSNs for 6 h. The nuclei of the RAW264.7 macrophages were stained with Hoechst 33,342. The phagocytosis and fluorescence signals of CCM@MSNs in macrophages were observed under a laser confocal fluorescence microscope (LCFM, TCS SP8 CARS, Leica Germany).

### Evaluation of CCM@MSNs targeting in vitro

OCI-LY10 cells (1 × 10^5^cells/well) were inoculated in culture dishes, and CCM vesicles labeled with DSPE-FITC were co-cultured with OCI-LY10 cells for 6 h, while OCI-LY10 nuclei were stained with OCI-LY10 Hoechst 33342. After washing with PBS three times, the image was observed under a LCFM.

### Cytotoxic effect of CCM@MSNs-ISOIM on OCI-LY10 in vitro

Cell viability was measured according to the instructions provided by CCK-8. A colony formation test was used to determine the inhibition rate of OCI-LY10 by CCM@MSNs-ISOIM. OCI-LY10 cells in logarithmic phase were collected and inoculated in 6-well plates, and the cell density was adjusted to 1 × 10^3^ cells/well. The cells were incubated in a cell incubator for 24 h. Following incubation, the cells were treated with various treatments and continued to be cultured for 12 days, changing the liquid every 3 days. After the formation of monoclonal colonies, the cells were fixed with methanol, stained with 0.2% crystal violet for 30 min, then rinsed with PBS five times and photographed. Single clones of more than 50 cells were counted.

### Cell Cycle, ROS, MMP and Apoptosis assay by flow cytometry in vitro

OCI-LY10 cells (5 × 10^5^ cells/well) were inoculated into 6-well plates and treated using different treatments for 48 h. After 48 h, the cells were collected and suspended in PBS after washing. Following PI staining, DNA content was measured via flow cytometry (FACS CantoTM II, BD, USA), and the cell cycle was analyzed. Fluorescent probe Rh123 was performed to detect the changes in level of mitochondrial membrane potential (MMP) by flow cytometry. The level of reactive oxygen species (ROS) in cells was detected by the ROS detection kit. In order to further determine the anti-tumor properties of CCM@MSNs-ISOIM in vitro, apoptosis was observed via Annexin V-FITC/PI apoptosis detection kit.

### Establishment of subcutaneous OCI-LY10 cell lymphoma model in nude mice

OCI-LY10 cells in a logarithmic growth phase were collected and resuscitated with PBS solution. The cells were resuscitated to 1 × 10^8^/mL, loaded into an EP tube and placed on crushed ice. About 0.2 mL cell suspension was absorbed with a 1 mL syringe, which was then inoculated subcutaneously after iodophor disinfection. After vaccination, the spirit, diet and defecation habits of nude mice were regularly observed.

### Evaluation of CCM@MSNs-ISOIM targeting in vivo

In order to evaluate CCM@MSNs-ISOIM targeting among tumor-bearing mice, Cy5-labeled CCM@MSNs and Cy5-labeled MSNs were compared and injected into tumor-bearing mice via tail vein. Using the Xenogen IVIS Lumina XR imaging system (Caliper Life Sciences, USA), the fluorescence signals at 6, 24 and 48 h after administration were detected.

### Evaluation of antitumor efficacy and toxicity of CCM@MSNs-ISOIM

When the tumor volume reached approximately 100 mm^3^, the experiment was divided into 4 groups (PBS, ISOIM, MSNs@ISOIM and CCM@MSNs-ISOIM), with 3 nude mice in each group. The dose of ISOIM has been factored into OCI-LY10 tumor-bearing mouse model, e.g. 10 mg of ISOIM/kg was used to treat the animal model of tumor-bearing mice [8]. Likewise, to draw comparison in the anti-tumor effects produced by MSNs@ISOIM, CCM@MSNs-ISOIM and free ISOIM, the concentration of ISOIM in MSNs@ISOIM and CCM@MSNs-ISOIM nanocomposites has been made identical to that of free ISOIM for the aforementioned research. The drugs were given by tail vein injection, once every 2 days, for a total of 4 times. The tumor length (L, mm), width (W, mm) and volume (V, mm^3^) were measured with an electronic Vernier caliper. The tumor volume was calculated by V=(LW^2^)/2. The tumor volume was measured through monitoring every 3 days, and the time curve of tumor volume was drawn. As the change in body weight is a sensitive index for drug toxicity and side effects, an electronic scale was also used to monitor the change in the body weight of the nude mice. The nude mice were weighed every 3 days, and the time curve depicting the weight changes of the nude mice was drawn. After the end of the aforementioned treatment, the nude mice were euthanized with anesthesia, and the tumor, heart, liver, spleen, lungs and kidneys were peeled off and fixed using a tissue fixation solution containing 4% polyformaldehyde for 24 h. The tumor and organ tissues of the nude mice were collected and were Hematoxylin and Eosin (H&E) stained to observe any histopathological changes. The blood of the nude mice was collected for hematological and biochemical analysis. Serum liver function was assessed using alanine aminotransferase (ALT) and aspartate aminotransferase (AST), while renal function was measured by creatinine (CRE) and blood urea nitrogen (BUN) and cardiac function was measured by creatine kinase (CK) and myoglobin (Myo). The levels of white blood cell (WBC), hemoglobin (HGB) and platelet (PLT) were also analyzed to observe hematological toxicity in vivo.

### TUNEL, Ki-67, MMP and ROS assays

Paraffin-embedded tissue samples were dewaxed, and the antigens were recovered. Apoptotic nuclei were detected using the TDT in situ apoptosis kit. The Ki-67 test kit was operated according to the manufacturer’s instructions. The morphology of the cells was observed under a light microscope, and its images were collected. MMP and ROS were detected via immunofluorescence staining of JC-1 and DCFH-DA, respectively. The nucleus was re-stained with DAPI, and LCFM was used to take the corresponding images.

### Western Blot analysis and immunofluorescence staining

After OCILY10 cells were treated with PBS, ISOIM, MSNs@ISOIM and CCM@MSNs-ISOIM, respectively, for 48 h, the above cells were collected and homogenized, after which the lysate was added at 4 ℃ for 30 min. The supernatant was extracted via centrifugation, and the protein concentration was determined by BCA. The protein contents of Pan-Cadherin, E-Cadherin, p53, caspase-3, caspase-9 and β-actin in the cells were observed through Western Blot analysis. OCI-LY10 tumor-bearing mice were treated with PBS, ISOIM, MSNs@ISOIM and CCM@MSNs-ISOIM, respectively, through tail vein once every 2 days for 4 times. After 21 days, the tumor tissues were collected. Paraffin embedded tumor tissue was deparaffinized for antigen repair, and immunofluorescence staining was carried out using anti-p53, Caspase-3 and caspase-9, according to the standard protocol. The tumor cells were observed and imaged under a fluorescence microscope.

### Statistical analysis

SPSS 16.0 software was used to conduct the statistical analysis, where data were expressed as mean ± SD. Differences between groups were assessed using one-way ANOVA, followed by Tukey’s post hoc test (*p < 0.05, **p < 0.01, and ***p < 0.001).

## Results

### Preparation and characterization of CCM@MSNs-ISOIM

**Fig. 2:**
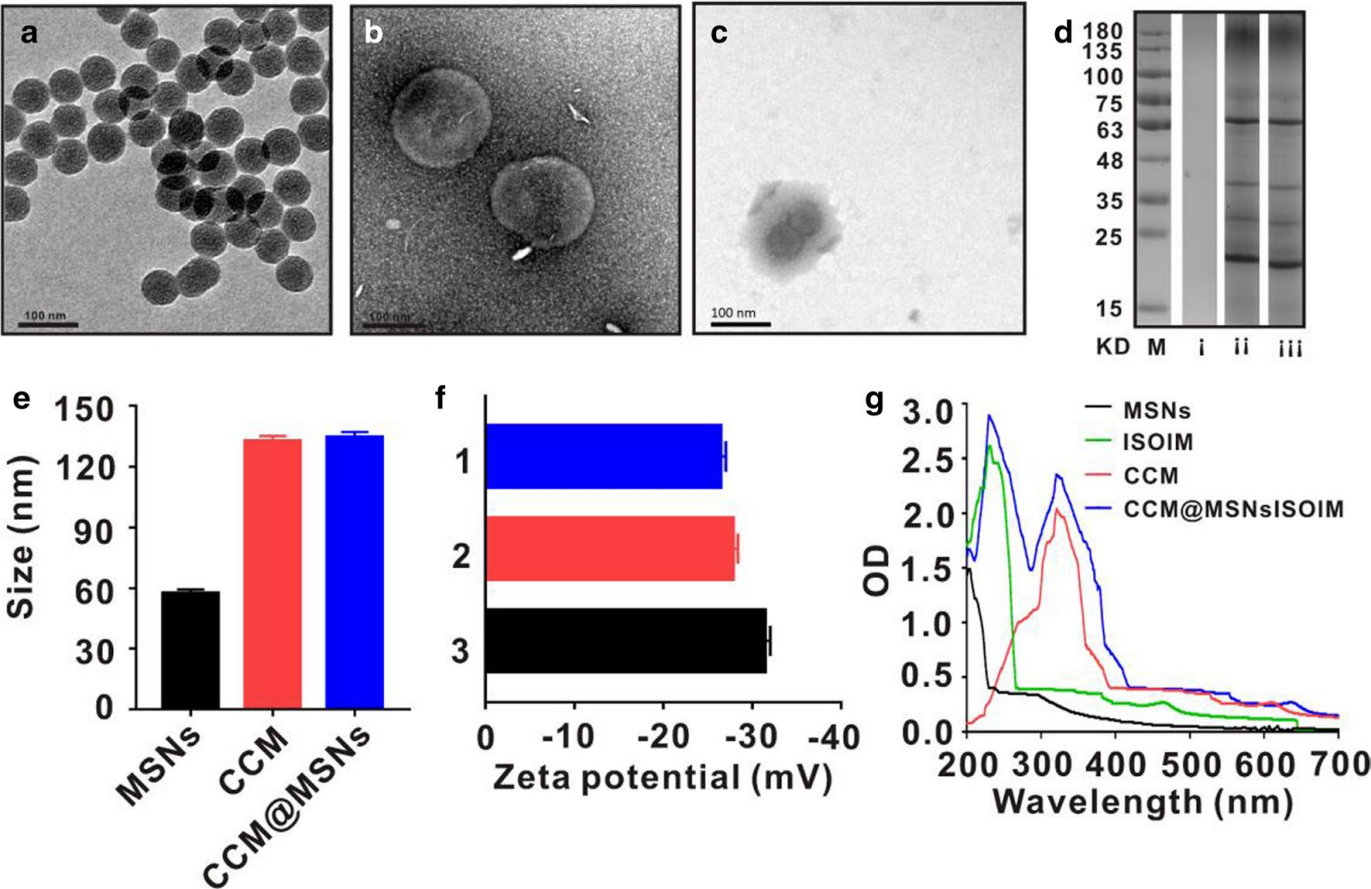
Characteristics of CCM@MSNs-ISOIM. TEM images: **a** MSNs, **b** CCM vesicles, **c** CCM vesicles camouflage MSNs. **d** SDS-PAGE protein analysis. (M) markers, (i) MSNs, (ii) CCM vesicles, and (iii) CCM vesicles camouflage MSNs. **e**, **f** The particle size and Zeta potential of (1) MSNs, (2) CCM vesicles and (3) CCM vesicles camouflaged MSNs, respectively. **g** UV–Vis spectra of ISOIM, MSNs, CCM vesicles and CCM@MSNs-ISOIM. Data are mean ± SD (n = 3)

First, ISOIM was loaded into MSNs to form MSNs@ISOIM, after which MSNs@ISOIM were coated with CCM vesicles using ultrasound to prepare the CCM@MSNs-ISOIM nanocomposites (Fig. 1). The transmission electron microscope (TEM) images showed that particle size uniformity and particle dispersion of MSNs were satisfactory (Fig. 2a). In addition, TEM also demonstrated that the membrane wraps the outer surface of the nanoparticles and forms a “shell core” structure, which signifies that the MSNs were successfully coated by the cancer cell membrane (Fig. 2b, c). Results from SDS-PAGE (Fig. 2d) showed that almost all CCM proteins in the CCM@MSNss nanocomplex were retained, and the particle size analyzer (Fig. 2e) illustrated that the particle size of MSNs was 57.6 ± 0.35 nm, while that of CCM@MSNs was 134.5 ± 0.96 nm, close to that of the cancer cell membrane (132.6 ± 1.12 nm). The potential of MSNs was − 31.4 ± 0.61 mV, while that of CCM@MSNs was − 26.2 ± 0.73 mV, similar to that of the cancer cell membrane (-27.8 ± 0.56 mV) (Fig. 2f). UV/vis spectra (Fig. 2g) depicted that the absorption peaks of CCM@MSNs-ISOIM were 321 nm, 202 nm and 230.5 nm, which were consistent with the absorption peaks of the CCM vesicles, MSNs and ISOIM, respectively, further confirming the CCM@MSNs-ISOIM’s successful construction.

### Drug loading and release

**Fig. 3:**
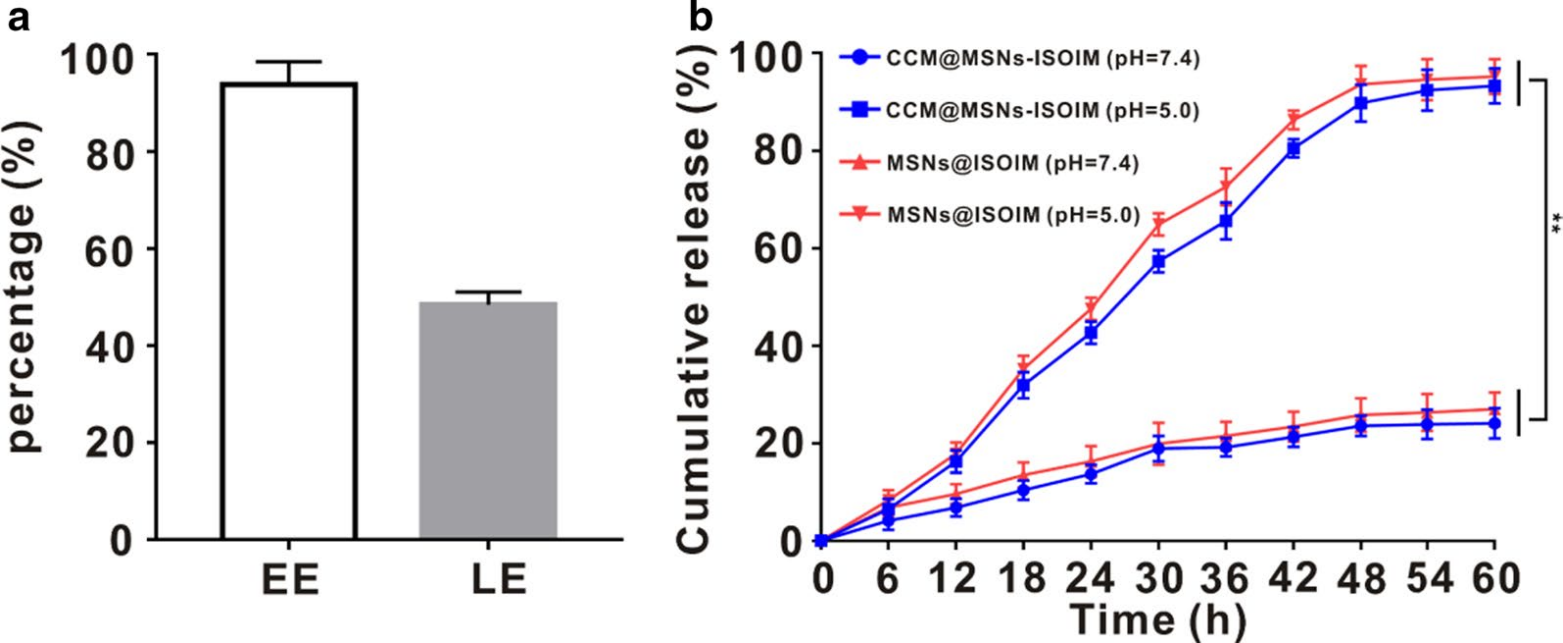
Drug loading and release. **a** EE and LE of MSNs. **b** The cumulative release rate of ISOIM from CCM@MSNs-ISOIM or MSNs@ISOIM at different pH values (7.4 and 5.0). Data are mean ± SD (n = 3), (* *p* < 0.05 and ** *p* < 0.01)

MSNs were used as a carrier for drug loading, hence, the entrapment efficiency (EE) and loading efficiency (LE) of ISOIM loaded on the CCM@MSNs-ISOIM nanocomplex were verified to be 93.8 ± 4.7% and 45.4 ± 2.6%, respectively (Fig. 3a). Based on mesoporous silica materials, pH-Responsive systems usually involve on/off capping or gating (by functional groups [31], polyelectrolytes [32] and ring-shaped compounds [33]) or host–guest interactions (electrostatic [34], covalent bonding [35] and coordination bonding [36]). Due to the complex procedures, it is often costly to prepare the pH responsive systems on a large scale. In order to improve the consequent adsorption and conjugation of molecules, it is usually necessary for the surface of mesoporous silica materials to be modified by introducing additional functional groups, which might put the further applications at risk for the pharmaceutical industry [37]. In this sense, it is of practical significance to develop a simple way to construct the pH-responsive drug delivery system using non-functionalized mesoporous silica materials [38]. Thus, in order to build up an efficient and economical drug delivery system, mesoporous silica nanoparticles (MSNs) are considered more appropriate to construct a simple drug delivery system through the direct host–guest interactions occurring between MSNs and drug molecules [39]. The in vitro ISOIM release characteristics of CCM@MSNs-ISOIM and MSNs-ISOIM were then evaluated (Fig. 3b). At pH 7.4, the ISOIM release rates of CCM@MSNs-ISOIM and MSNs-ISOIM within 60 h were 24.1 ± 3.1% and 27.0 ± 3.4%, respectively, whereas at pH 5.0, the release rates of CCM@MSNs-ISOIM and MSNs-ISOIM were 93.3 ± 3.6% and 95.2 ± 2.9%, respectively. The above results show that the release rate of ISOIM at pH 5.0 is faster than that at pH 7.4. According to the in vitro drug loading and release experiments, MSNs were found to be efficient drug carriers. The acidic environment of the tumor [40] is conducive to the release of ISOIM from CCM@MSNs-ISOIM, and MSNs possess certain characteristics in low pH value sensitivity.

### Biocompatibility studies of CCM@MSNs-ISOIM in vitro

**Fig. 4:**
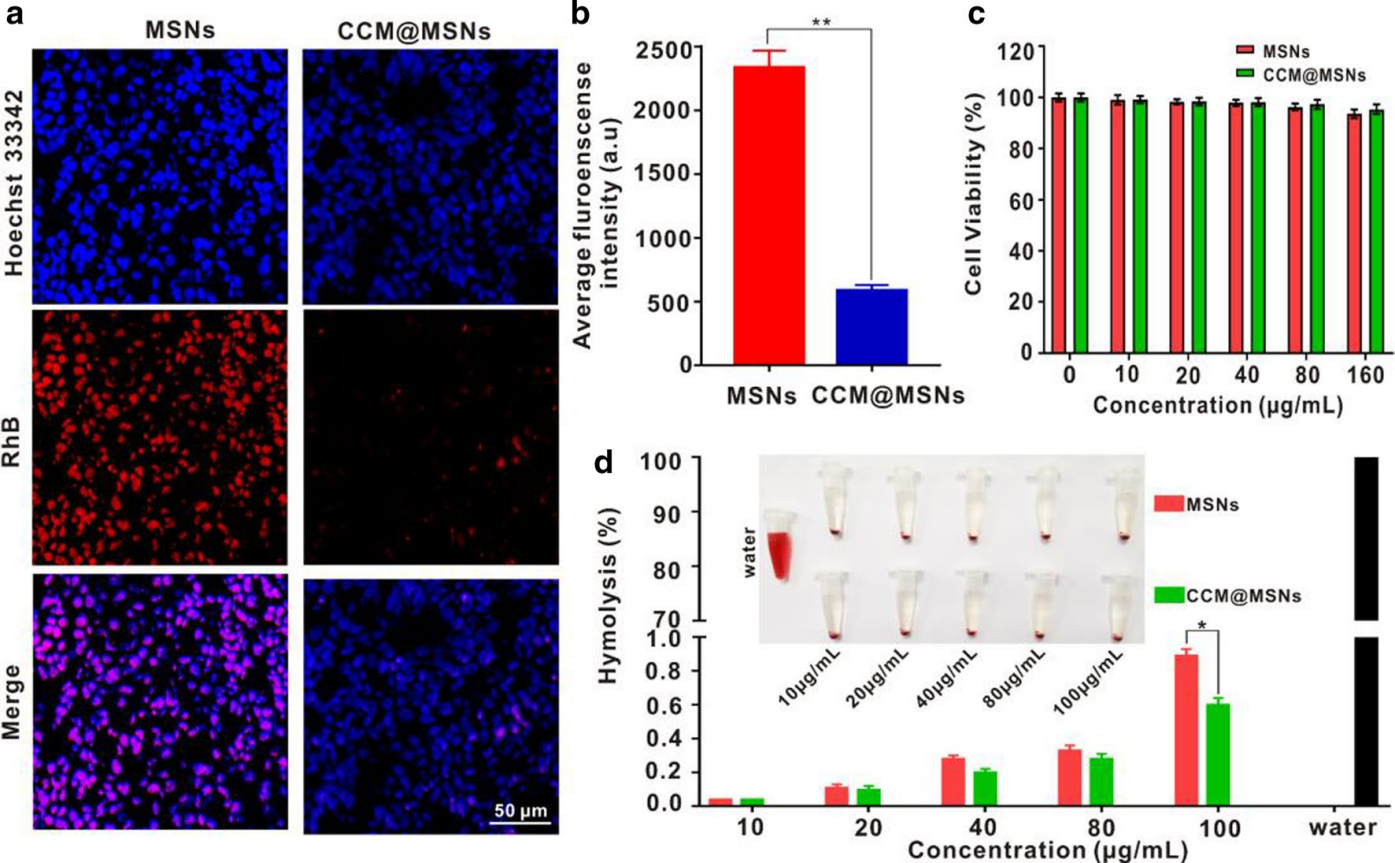
Biocompatibility of CCM@MSNs-ISOIM. **a** LCFM images of RAW264.7 macrophages co-cultured with MSNs-RhB and CCM@MSNs-RhB for 6 h. Scale bar: 50 μm. **b** The fluorescence intensity of cells treated with MSNs-RhB and CCM@MSNs-RhB was quantified by fluorophotometer. **c** The cell viability of OCI-LY10 cells treated with different concentrations of MSNs and CCM@MSNs. **d** Hemolysis rate of red blood cells in different concentrations of MSNs and CCM@MSNs. Data are mean ± SD (n = 3), (* *p* < 0.05 and ** *p* < 0.01)

The phagocytosis and hemolysis tests verified the biocompatibility of CCM@MSNs-ISOIM nanocomposites in vitro. CCM@MSNs and MSNs were labeled with RhB (red fluorescence), respectively, after which RAW264.7 macrophages were treated with CCM@MSNs-RhB and MSNs-RhB for 6 h, respectively. When macrophages are treated with MSNs-RhB, most macrophages demonstrated diffuse red fluorescence, indicating that macrophages strongly phagocytose MSNs (Fig. 4a, b). In contrast, a weak degree of red fluorescence was observed by CCM@MSNs-RhB, indicating that CCM vesicles effectively inhibited the phagocytosis of macrophages. Accordingly, CCM vesicles were observed to enable the nanomaterials’ ability of immune escape, reducing the clearance rate. The in vitro toxicity of CCM@MSNs and MSNs were also evaluated through the detection of cell viability as well as the hemolysis rate of erythrocytes. Next, the cell viability of OCI-LY10 cells was determined by CCK-8. Figure 4c depicts the cell survival rate of OCI-LY10 cells incubated with a series of concentrations of MSNs or CCM@MSNs for 24 h, of which the survival rate of OCI-LY10 cells treated with 100 µg/mL CCM@MSNs was found to be as high as 90%. As illustrated in Fig. 4d, no obvious hemolysis was evident in erythrocytes treated with CCM@MSNs and MSNs. Additionally, the hemolysis rate caused by CCM@MSNs was less than 1%, lower than that caused by MSNs. Therefore, the obtained results further indicate that CCM@MSNs possesses excellent biocompatibility.

### Verification of targeting and antigen functionalization of cancer cell membranes in vitro

**Fig. 5:**
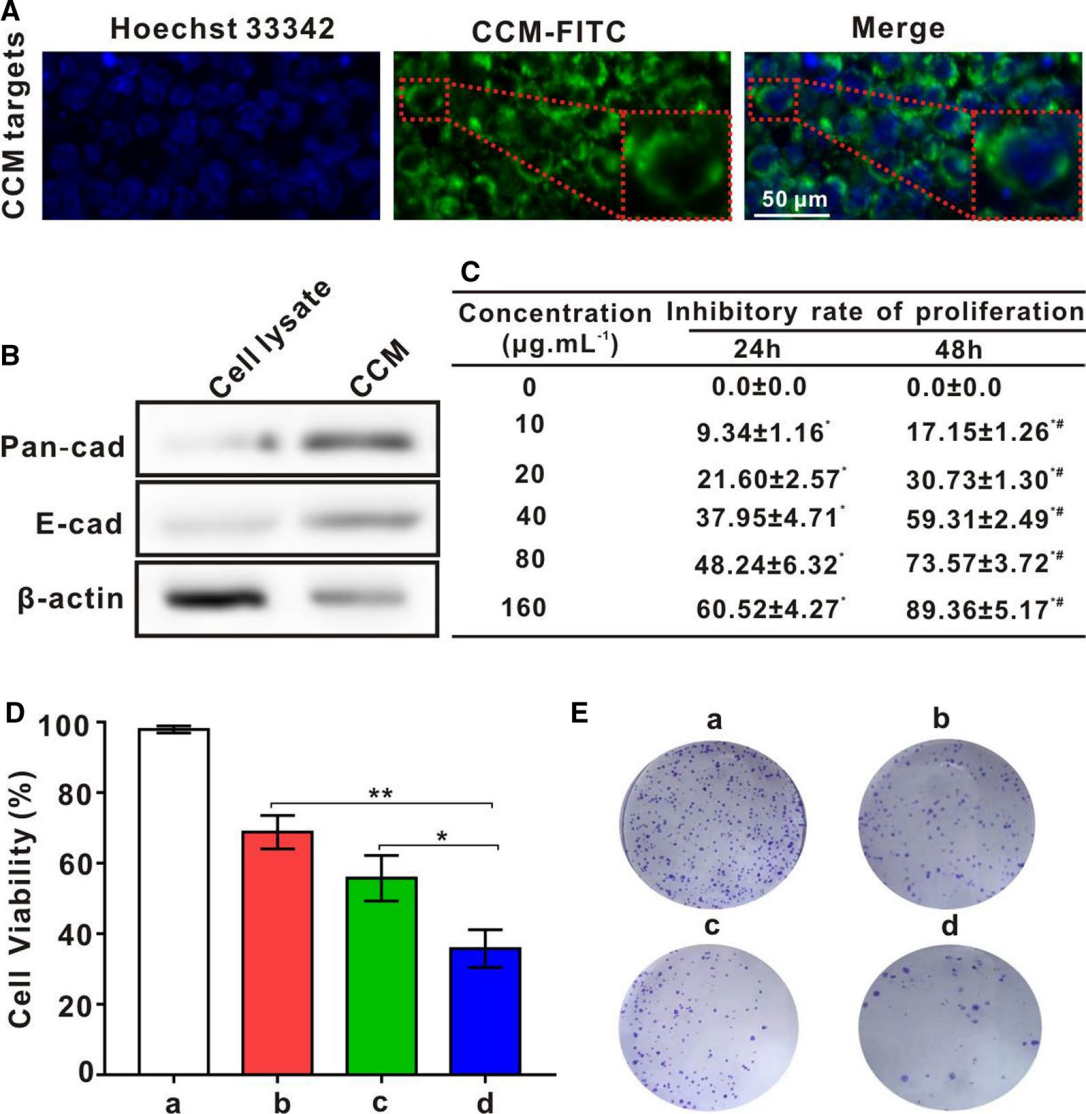
Study on the targeting of CCM and the anti-tumor effect of CCM@MSNs-ISOIM in vitro. **A** The LCFM images of DSPE-FITC-labeled CCM vesicles were co-cultured with OCI-LY10 cells for 6 h. **B** Detection of membrane specific protein Pan-cadherin and E-cadherin by Western blotting. **C** Proliferation inhibition rate of OCI-LY10 cells treated with different concentrations of ISOIM for 24 h and 48 h. (* p < 0.05 compared with 0 μg. mL^−1^, ^#^p < 0.05 compared with 24 h). **D** Survival rate of OCI-LY10 cells treated with a) PBS, b) ISOIM, c) MSNs@ISOIM and d) CCM@MSNs-ISOIM for 48 h, respectively. **E** Representative images of colony formation of OCI-L10 cells treated with a) PBS, b) ISOIM, c) MSNs@ISOIM and d) CCM@MSNs-ISOIM for 14 days. Data are presented as the mean ± SD (n = 3). (* *p* < 0.05 and ** *p* < 0.01)

In order to determine the targeting of CCM vesicles for lymphoma cells, DSPE-FITC (green fluorescence) was used to label the CCM vesicles. The nucleus of OCI-LY10 was stained blue using Hoechst 33,342. As shown in Fig. 5A, DSPE-FITC-CCM vesicles gathered around the nucleus of OCI-LY10, indicating that CCM vesicles may adhere to OCI-LY10 cells with obvious active targeting abilities. In order to verify the existence of specific membrane antigens on CCM vesicles, cancer cell membrane protein markers and intracellular markers were analyzed via Western Blot. As shown in Fig. 5B, specific cancer cell membrane marker pan-cadherin and E-cadherin were observed [24, 41]. However, as an indicator of cytoplasmic protein, cytoskeleton protein β-actin is mostly found in the bands of tumor cell lysate, where cancer cell membrane extract contents are less. Therefore, after hypotonic lysis and gradient centrifugation, the cytoplasmic components of lymphoma cells were basically removed, whereas the proteins on the lymphoma cell membrane remained on its surface.

### Effect of CCM@MSNs-ISOIM on the survival rate of OCI-LY10 cells in vitro

According to the results of Fig. 5C, the cell proliferation inhibition rate of OCI-LY10 cells treated with different concentrations of ISOIM for 24 and 48 h were higher than that of cells without ISOIM treatment, and the rate increased with a rise in drug dose, suggesting that the inhibitory effect of ISOIM on the proliferation of OCI-LY10 cells was dose-dependent. For times under 48 h, the cell proliferation inhibition rate gradually increased as time progressed, while the rate at 48 h was higher than that at 24 h (P < 0.05). The IC50 score of OCI-LY10 cells at 24 h and 48 h were 87 µg/mL and 33 µg/mL, respectively. Therefore, the concentration of 33 µg/mL of free ISOIM was chosen for the follow-up experimental study. At present, the clinical dosage forms of traditional Chinese medicine mainly stay in the traditional dosage forms such as ointment, soup, pill and powder, while the anti-tumor active components contained in some traditional Chinese medicine show such drawbacks as poor water solubility, low bioavailability and low specificity distribution. Besides, most of them are administered orally, and the active, ineffective and even toxic components of the preparation are also introduced into the body [42]. With the drug encapsulated in the nano-drug delivery system, the active components of traditional Chinese medicine can penetrate the reticuloendothelial system, which not only improves the solubility of the drug, but also enhance the performance of the drug in tumor targeting through passive and active targeting. In the meantime, the tumor microenvironment can be taken advantage of to regulate the drug release performance. In this way, the drug can be released either synchronously or sequentially, thus enhancing the curative effect of the drug on the tumor, mitigating the side effects of the drug on the normal tissue, and reducing the toxic and side effects caused by clinical treatment [43]. As mentioned above, in order to address poor water solubility, short half-life, poor stability, low bioavailability and the toxicity of ISOIM, nano-preparation (MSNs@ISOIM and CCM@MSNs-ISOIM) was modified for comparison with free ISOIM to further demonstrate the advantages of nanotechnology and biomimetic nano-modification technology [44]. In order to verify the effects of ISOIM, MSNs@ISOIM and CCM@MSNs-ISOIM in the same experiment, the concentration of ISOIM in both MSNs@ISOIM and CCM@MSNs-ISOIM was set to 33 µg/mL. As depicted in Fig. 5D, compared to the PBS group, the cell survival rates of the ISOIM, MSNs@ISOIM and CCM@MSNs-ISOIM groups were 68.83 ± 4.72%, 55.80 ± 6.44% and 35.85 ± 5.36%, respectively. In the same experiment, the effects of ISOIM, MSNs@ISOIM and CCM@MSNs-ISOIM were compared, where the ISOIM concentration of MSNs@ISOIM and CCM@MSNs-ISOIM was found to be 33 µg/mL. The cell survival rate of the MSNs@ISOIM and CCM@MSNs-ISOIM groups were significantly lower than that of the ISOIM group. In short, CCM@MSNs-ISOIM was observed to possess the most significant effects of anti-lymphoma. Similarly, compared to the PBS group, both the ISOIM and CCM@MSNs-ISOIM groups inhibited colony formation of OCI-LY10 cells, and the inhibitory effect of the CCM@MSNs-ISOIM group on OCI-LY10 clone formation was found to be the most significant (Fig. 5E).

### Effects of CCM@MSNs-ISOIM on cell cycle, apoptosis, reactive oxygen species (ROS), mitochondrial membrane potential (MMP) and apoptosis proteins

**Fig. 6:**
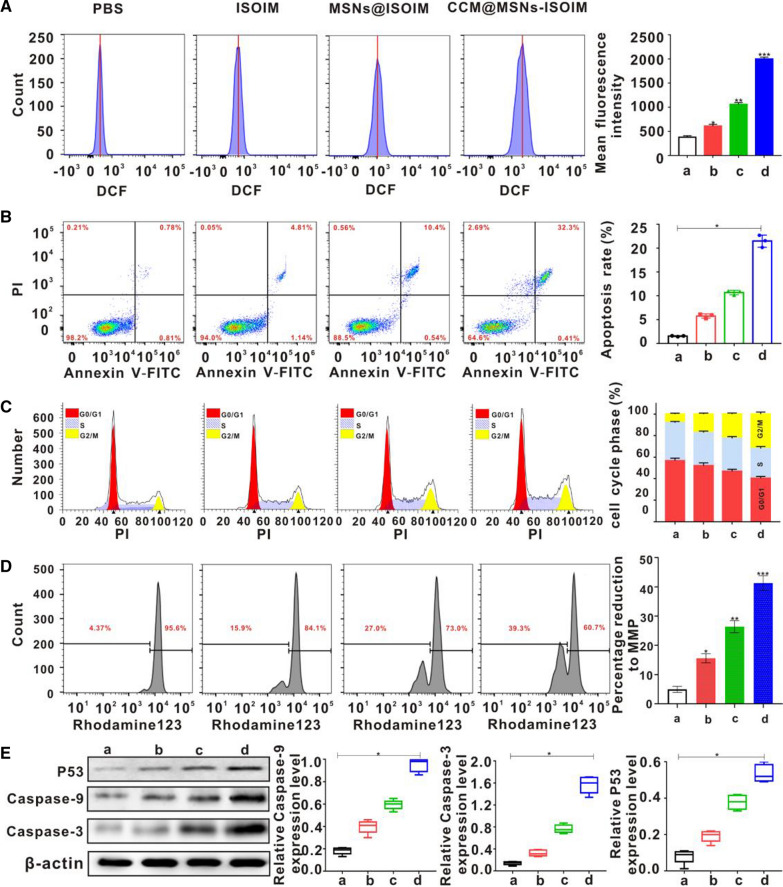
Anti-lymphoma effect of CCM@MSNs-ISOIM in vitro was detected by flow cytometry and detection of apoptosis-related protein expression by WB. OCI-LY10 cells were treated with a) PBS, b) ISOIM, c) MSNs@ISOIM and d) CCM@MSNs-ISOIM for 48 h, respectively. **A** ROS level of OCI-LY10 cells was detected by flow cytometry after 48 h. **B** Flow cytometry was used to detect the apoptosis of OCI-LY10 cells after 48 h. **C** Flow cytometry was used to detect the changes of cell cycle of OCI-LY10 cells after 48 h. **D** Flow cytometry was used to detect the changes of MMP in OCI-LY10 cells after 48 h. **E** The expression of apoptosis-related proteins p53, Caspase-9 and Caspase-3 were detected by Western blotting. Data are presented as the mean ± SD (n = 3). (* *p* < 0.05, ** *p* < 0.01, and *** *p* < 0.001)

After OCI-LY10 cells were treated with PBS, ISOIM, MSNs@ISOIM and CCM@MSNs-ISOIM for 24 h, DCFH-DA was used as a fluorescence probe to detect the levels of intracellular ROS. The results of the flow cytometry in Fig. 6A demonstrated that ROS level in the ISOIM, MSNs@ISOIM and CCM@MSNs-ISOIM groups were significantly higher than that in the PBS group, where CCM@MSNs-ISOIM was observed to be the most significant. Accordingly, CCM@MSNs-ISOIM was suggested to have the strongest effect on ROS production in OCI-LY10 cells. The results of the Annexin V-FITC/PI double staining flow cytometry showed that compared to PBS, the number of living cells decreased significantly, while the number of apoptosis cells increased significantly following ISOIM, MSNs@ISOIM and CCM@MSNs-ISOIM intervention for 48 h, from 5.76 ± 0.46% and 10.70 ± 0.48 to 21.48 ± 1.27% (Fig. 6B). Moreover, CCM@MSNs-ISOIM was confirmed to induce apoptosis in OCI-LY10 cells. After OCI-LY10 cells were treated with ISOIM, MSNs@ISOIM and CCM@MSNs-ISOIM for 48 h, the proportion of cells in the G2/M phase was 17.15 ± 0.9%, 22.50 ± 1.02% and 31.93 ± 2.64%, respectively. Compared to the PBS group, the above results showed a gradually increasing trend in the G2/M phase, whereas the cells in the G0/G1 phase showed a relatively decreasing trend. Hence, CCM@MSNs-ISOIM was suggested to induce cell cycle arrest of OCI-LY10 cells in the G2/M phase (Fig. 6C). Furthermore, the effects of ISOIM, MSNs@ISOIM and CCM@MSNs-ISOIM on MMP of OCI-LY10 cells by flow cytometry with JC-1 staining were evaluated. As shown in Fig. 6D, the percentage of reduction to mitochondrial membrane potential reached 41.3 ± 2.5% after treatment with CCM@MSNs-ISOIM nanocomplex, which was higher compared with ISOIM group (15.6 ± 1.6%). To this effect, CCM@MSNs-ISOIM was inferred to damage the mitochondria of cells, leading to a decrease in MMP. Subsequently, the effects of CCM@MSNs-ISOIM on the level of apoptosis-related proteins were observed via Western Blotting analysis (WB). Compared to PBS, ISOIM and MSNs@ISOIM, CCM@MSNs-ISOIM significantly upregulated the expression of p53, caspase-9 and caspase-3 in OCI-LY10 cells (Fig. 6e). According to the above results, after CCM@MSNs-ISOIM acts on OCI-LY10 cells in vitro, it was concluded that the induced ROS blocks the cell cycle and reduces MMP to promote the release of tumor suppressor gene p53 and caspase proteins, resulting in OCI-LY10 apoptosis.

### Verification of CCM@MSNs-ISOIM targeting and its anti-tumor effects in vivo

**Fig. 7:**
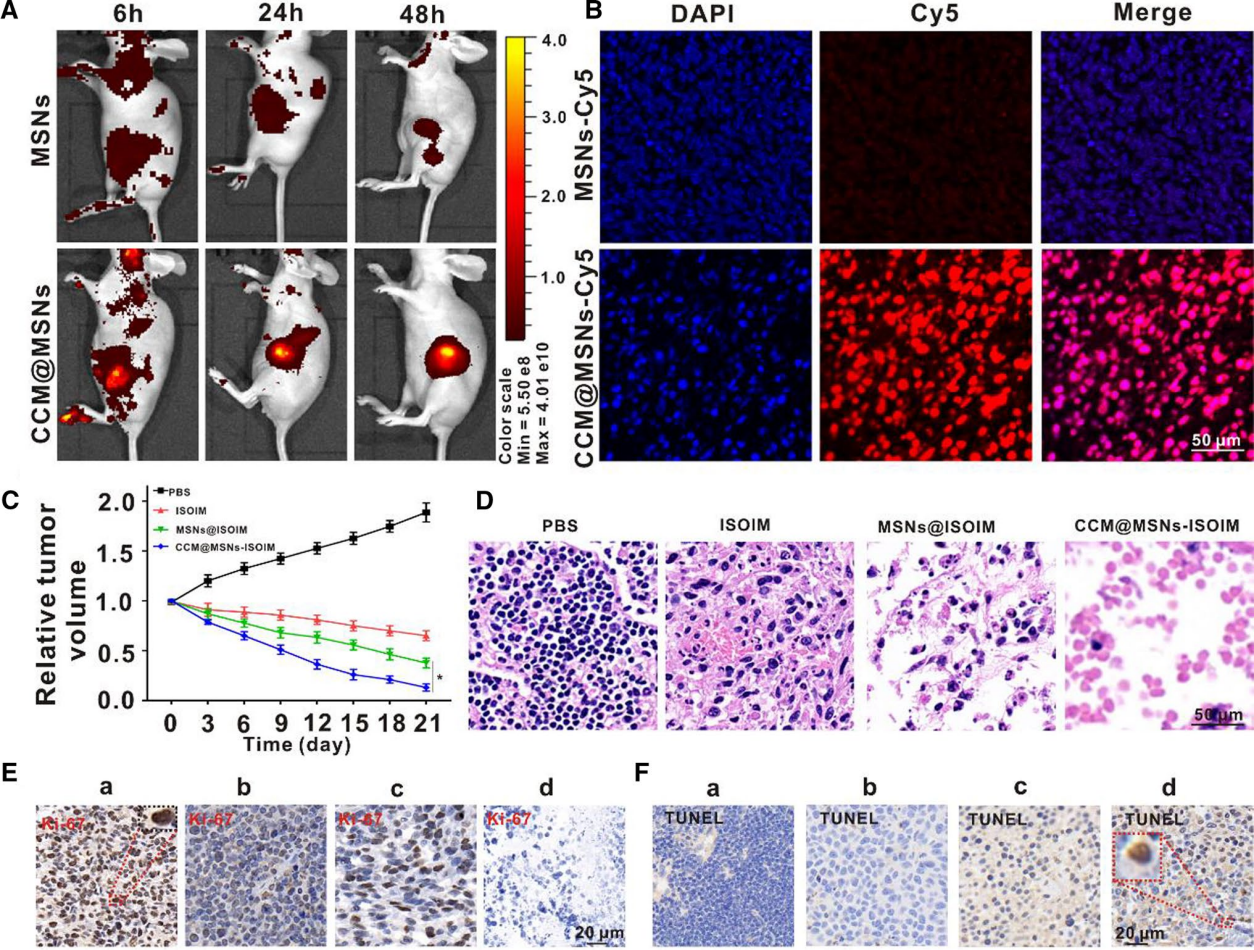
Verification of CCM@MSNs-ISOIM targeting and its anti-tumor effect in vivo. **A** The fluorescence images in vivo were observed at 6 h, 24 h and 48 h after MSNs-Cy5 and CCM@MSNs-Cy5, respectively, were injected into the tail vein of OCI-LY10 tumor-bearing mice. **B** MSNs-Cy5 and CCM@MSNs-Cy5 were given to the caudal vein respectively, and the fluorescence imaging of the tumor tissue of tumor-bearing mice was observed for 48 h. Scale bar: 50 μm. **C** Curve changes of tumor volume in OCI-LY10 tumor-bearing mice during treatment. **D** Different treatments were given to the caudal vein, and the H&E staining images of tumor tissues were observed on the 21st day. Scale bar: 50 μm. **E**, **F** Ki-67 and TUNEL were detected on the 21st day after a) PBS, b) ISOIM, c) MSNs@ISOIM and d) CCM@MSNs-ISOIM treatment by tail vein, respectively. Scale bar: 20 μm. Data are presented as the mean ± SD (n = 3). (* *p* < 0.05)

In vitro experiments have confirmed that CCM@MSNs are able to target tumor cells and possess the ability of immune escape, therefore, they may accumulate at the tumor site. To test this hypothesis in vivo, Cy5 was used to label CCM@MSNs and MSNs, respectively, with Cy5-MSNs as the control group. Their biological distribution in vivo was then evaluated using near-infrared fluorescence imaging. As shown in Fig. 7A, compared to Cy5-MSNs, the fluorescence intensity of the tumor site gradually increased following tail vein injection of CCM@MSNs-Cy5, indicating that CCM@MSNs accumulated more in the tumor than MSNs. Furthermore, 48 h after intravenous administration, the retention of CCM@MSNs in tumor tissue was found to be significantly higher than that of MSNs. In addition, bright red fluorescence was observed in the tumor tissue treated with CCM@MSNs-Cy5, which was more obvious than that in MSNs-Cy5 (Fig. 7B), indicating that CCM@MSNs could efficiently deliver drugs. The above results demonstrate that CCM@MSNs nanocomposites have the active tumor targeting abilities as well as a high efficiency in drug delivery and drug release.

Afterward, an anti-tumor test in vivo was carried out on OCI-LY10 tumor-bearing nude mice using the CCM@MSNs-ISOIM nanocomplex. When the tumor tissues were treated with PBS, ISOIM, MSNs@ISOIM and CCM@MSNs-ISOIM respectively, compared to the free ISOIM, both MSNs@ISOIM and CCM@MSNs-ISOIM were observed to significantly inhibit tumor growth, of which CCM@MSNs-ISOIM had the most significant inhibitory effect (Fig. 7C). Simultaneously H&E staining showed that the number of necrotic cells in the CCM@MSNs-ISOIM group was more than that in the other groups (Fig. 7D), further suggesting that CCM@MSNs-ISOIM possesses better effects in anti-lymphoma than that of the free ISOIM.

CCM@MSNs-ISOIM can block the OCI-LY10 cell cycle and inhibit cell proliferation in vitro, hence, Ki-67 immunohistochemical staining was used to detect cell proliferation in vivo [45]. As shown in Fig. 7E, the number of Ki-67 positive cells (brown) in the CCM@MSNs-ISOIM group was found to be significantly less than that in the other groups, suggesting that CCM@MSNs-ISOIM most significantly inhibited the proliferation of lymphoma cells. In order to verify the pro-apoptotic effect of CCM@MSNs-ISOIM in vivo, TUNEL staining was utilized to detect apoptosis [46]. As shown in Fig. 7F, nuclear staining of tumor cells in CCM@MSNs-ISOIM group was found to be positive (brown), which was significantly higher than that in the ISOIM group. The results of TUNEL were consistent with the apoptosis of lymphoma cells induced by CCM@MSNs-ISOIM in vitro.

### Anti-lymphoma mechanism of CCM@MSNs-ISOIM

**Fig. 8:**
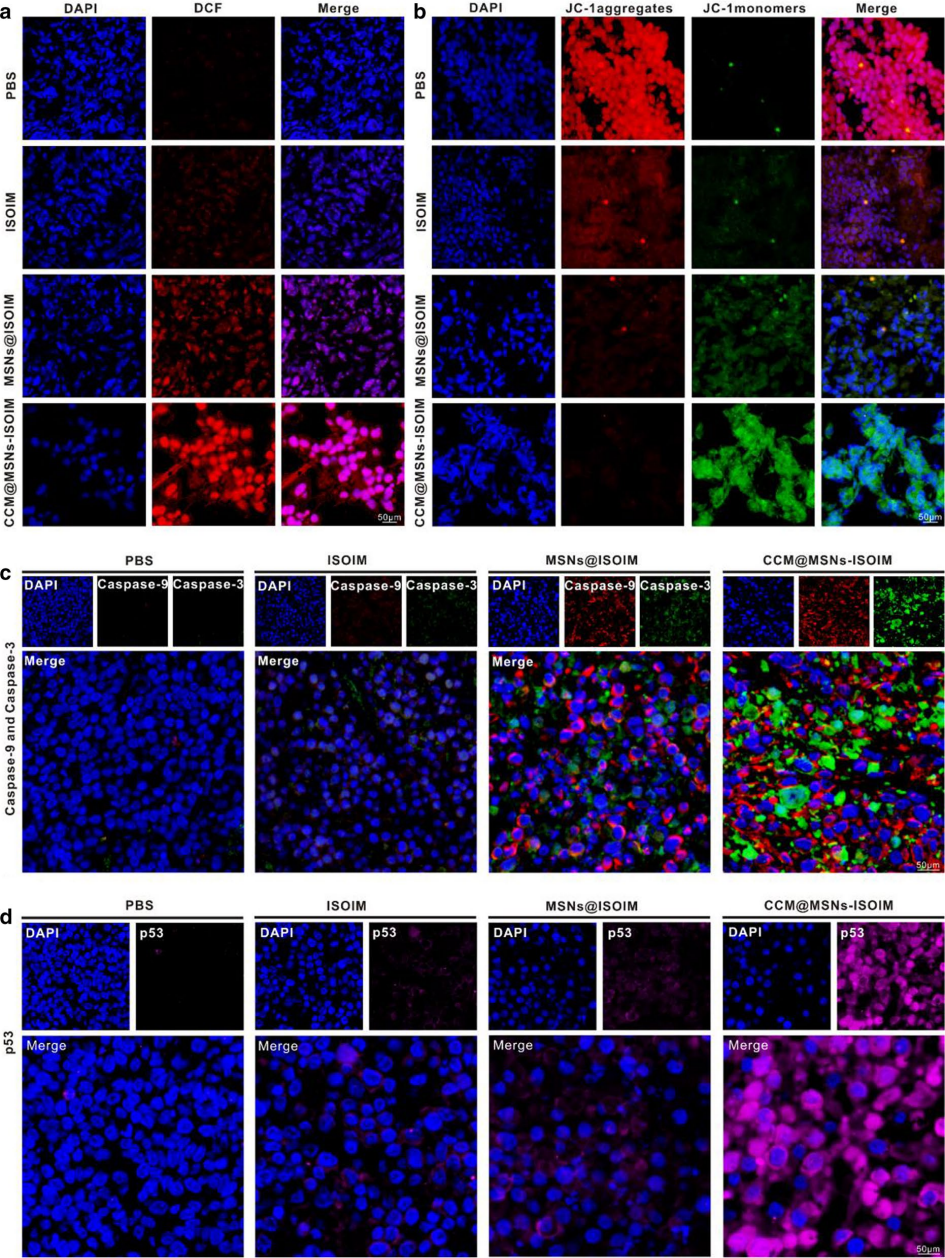
Immunofluorescent staining of tumor tissues. **a**, **b** Different treatments were given, and the tumor tissues were analyzed by ROS and MMP on the 21st day. Scale bar: 50 μm. **c**, **d** The LCFM images of the tumor area were taken after different treatments. Red: Caspase-9, green: Caspase-3, pink: p53, blue: nucleus. Scale bar: 50 μm

Apoptosis is one of the main ways in inhibiting the growth of tumor cells, and mitochondria are the most important organelles in the regulation of this process [47]. In addition to supplying energy to cells, mitochondria produce ROS, an apoptosis-inducing signal molecule [48]. ROS can directly trigger the opening of mitochondrial permeability transition pores and decrease the mitochondrial membrane potential, resulting in the activation of the mitochondrial Caspase-dependent apoptosis pathway [49, 50]. CCM@MSNs-ISOIM has a specific and powerful anti-tumor outcome in vitro, producing ROS to block the cell cycle while reducing MMP to activate apoptosis-related proteins, which promotes the mitochondrial apoptosis pathway against lymphoma. In order to verify whether CCM@MSNs-ISOIM also inhibits tumors via mitochondrial apoptosis pathway in vivo, the expression levels of ROS, MMP, Caspase-9 and Caspase-3 were detected. As shown in Fig. 8a, after the tumor was treated with PBS, ISOIM, MSNs@ISOIM and CCM@MSNs-ISOIM for 21 days, the red fluorescence signal in the tumor tissue section of the CCM@MSNs-ISOIM group was most evident, indicating that CCM@MSNs-ISOIM produced more ROS than ISOIM alone. Then, a small amount of green fluorescence was observed in the tumor tissue sections of the ISOIM group, whereas diffuse green fluorescence was observed in the tumor tissue sections of the CCM@MSNs-ISOIM group, suggesting that, compared to the other groups, CCM@MSNs-ISOIM contributed to the most significant decrease in MMP (Fig. 8b). By conducting an immunofluorescence assay to detect the expression of apoptosis protein Caspase-9 and Caspase-3, red (Caspase-9) and green (Caspase-3) fluorescence of the tumor tissue slices in the CCM@MSNs-ISOIM group depicted a strong level of fluorescence, while that of Caspase-9 and Caspase-3 in the other groups were significantly weaker, indicating that these two genes were highly expressed in tumor tissues treated with CCM@MSNs-ISOIM (Fig. 8c). P53 protein acts as the guardian of the genome while playing an important role in regulating cell proliferation [51]. Cellular stress like DNA damage and carcinogenic signals can activate the p53 tumor suppressor pathway and coordinate the transcriptional response of hundreds of genes [52]. The study further found that p53 activation induces DNA repair or apoptosis by initiating multiple pathways [53]. In view of the important role of p53 protein activation in cell cycle regulation, the effect of CCM@MSNs-ISOIM on p53 protein activation in NHL was discussed. Figure 8d showed that the fluorescence intensity (pink) of p53 in the CCM@MSNs-ISOIM group was more significant than that in the other groups, indicating that CCM@MSNs-ISOIM enhanced the expression of the p53 protein. CCM@MSNs-ISOIM may also induce DNA damage by inducing G2/M phase arrest, promoting the activation of p53 protein, which further leads to apoptosis of lymphoma cells. The corresponding results suggest that CCM@MSNs-ISOIM induces apoptosis of lymphoma through the G2/M/p53 and mitochondrial apoptosis pathways.

### Study on the biosafety of CCM@MSNs-ISOIM in vivo

**Fig. 9:**
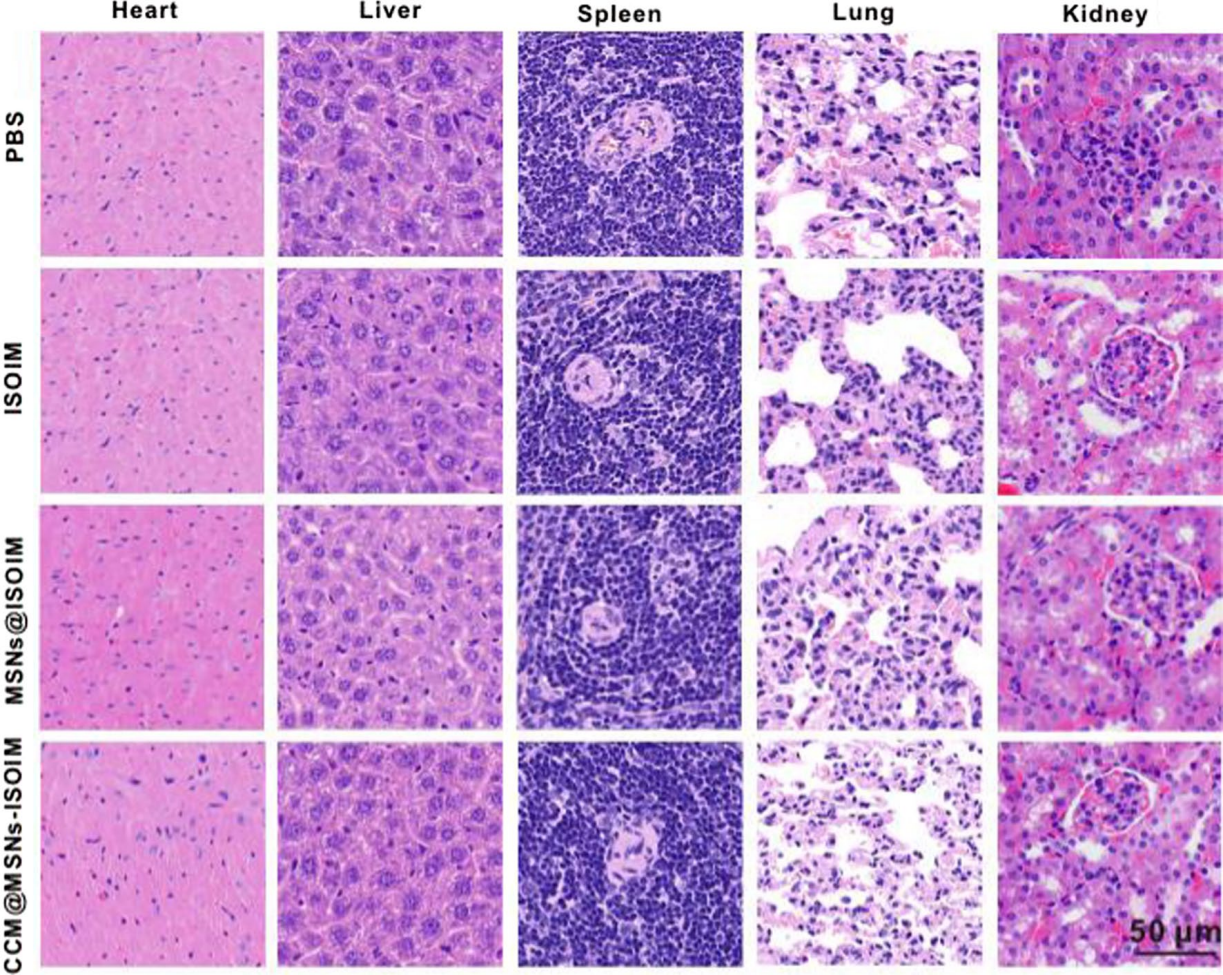
In vivo biological safety study. Nude mice were treated with PBS, ISOIM, MSNs@ISOIM and CCM@MSNs-ISOIM for three weeks, and the main organs were stained with H&E. Scale bar: 50 μm

**Fig. 10:**
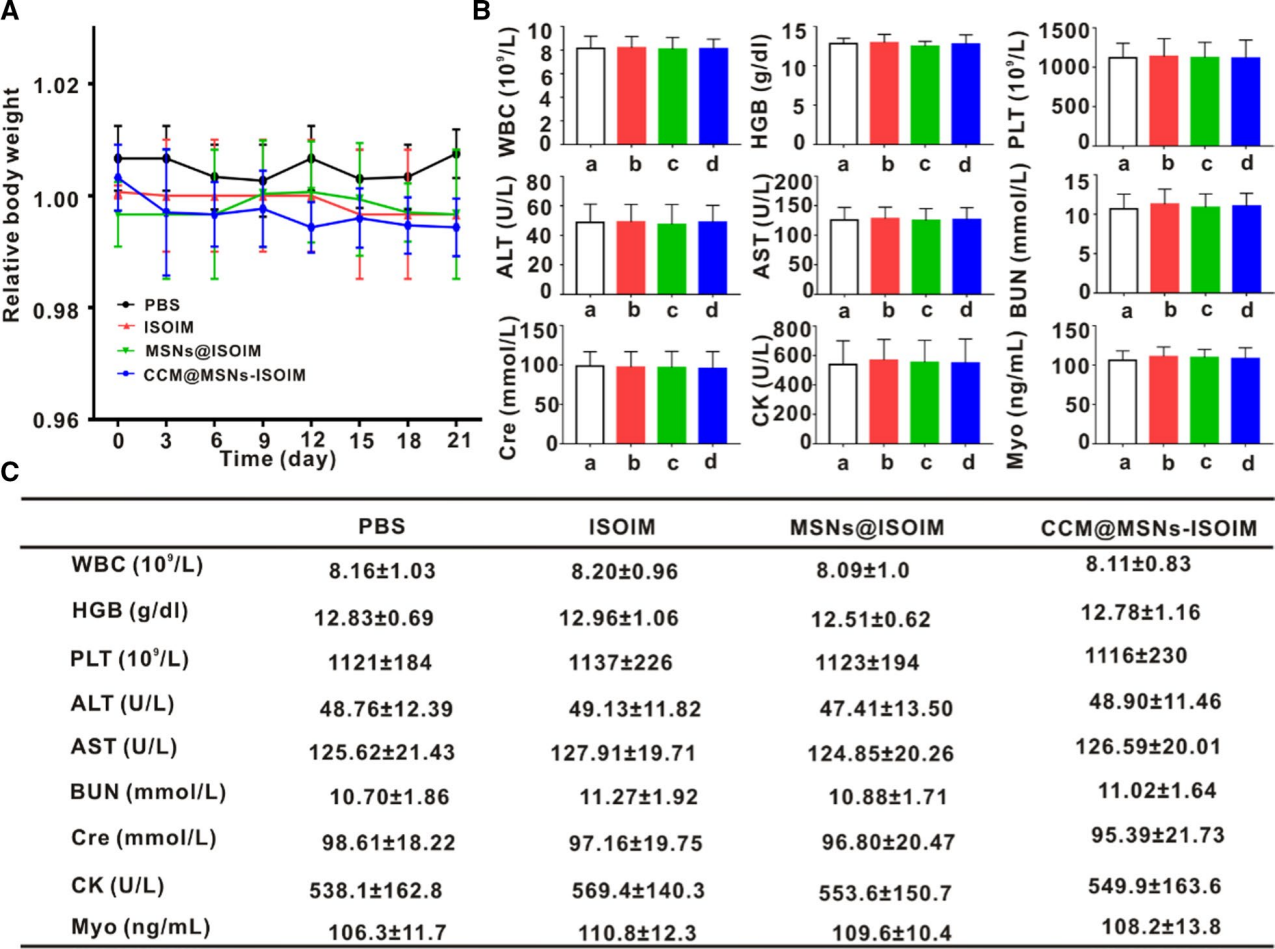
Body weight, blood routine and blood biochemical analysis of nude mice. **A** The change curve of body weight of OCI-LY10 tumor-bearing mice during treatment. **B** The nude mice were euthanized on the 21st day after PBS, ISOIM, MSNs@ISOIM and CCM@MSNs-ISOIM, respectively, were injected into the tail vein, and the blood routine indexes (WBC, HGB, and PLT) and blood biochemical indexes (ALT, AST, BUN, Cre, CK and Myo) were detected. **C** A table to summarize all data in **B** by putting the respective numbers. Data are presented as mean ± SD (n = 3)

To verify whether CCM@MSNs-ISOIM has potential toxicity in vivo, the toxicity of PBS, ISOIM, MSNs@ISOIM and CCM@MSNs-ISOIM were evaluated via tail vein injection in nude mice. A histological analysis was then carried out and compared to the PBS group, which demonstrated no significant pathological damage in the major organs of the nude mice treated with CCM@MSNs-ISOIM for 3 weeks (Fig. 9). In addition, no significant abnormal changes in the body weight of the nude mice were present in the above four groups within 3 weeks (Fig. 10A). Furthermore, no abnormal changes were present in WBC, HGB, PLT, ALT, AST, BUN, CRE, CK and Myo in each group (Fig. 10B, C), signifying that CCM@MSNs-ISOIM is non-toxic and possesses good biocompatibility.

## Discussion

Searching for effective antineoplastic drugs remains the focus of anti-tumor research. Plants usually contain a variety of anti-tumor compounds, and many plant extracts have been used in the treatment of cancer such as vincristine, paclitaxel and topotecan [54–56]. ISOIM is an active compound extracted from Chinese herbal medicine, which has been shown to possess anti-tumor properties in gastric, lung and ovarian cancer cells [9]. Among other malignancies, NHL has a high incidence and strong heterogeneity. Chemotherapy is commonly used to treat this disease at present, however, patients have different clinical outcomes due to their individual differences, which confers a certain impact on the quality of life for patients [57]. In order to discover novel therapies in the treatment of lymphoma, complementary and alternative medicine may shift its focus to traditional Chinese medicine. Certain traditional Chinese medicines have been shown to induce apoptosis in lymphoma cells [58–60]. However, most effective components in traditional Chinese medicine have various issues such as low water solubility, poor stability, rapid elimination in vivo, and high variation in pharmacokinetics [61]. To this effect, the emergence of nanotechnology has reduced such problems [62]. In this paper, MSNs-loaded ISOIM was used as the nano-core (MSNs@ISOIM), while CCM was used to wrap MSNs@ISOIM in order to construct a novel anti-tumor nano-traditional Chinese medicine (CCM@MSNs-ISOIM).

In the present study, CCK-8 detection showed that ISOIM inhibits the proliferation of OCI-LY10 cells in a dose and time-dependent manner. During the CCK-8 experiment, the IC50 value (33 µg/mL) showed that the same concentration of ISOIM, CCM@MSNs-ISOIM may significantly inhibit the proliferation of OCI-LY10 compared to the free ISOIM (P < 0.05). Cell clone formation assays also confirmed that CCM@MSNs-ISOIM inhibits the proliferation of OCI-LY10 cells. AnnexinV-FITC/PI double staining flow cytometry was also performed along with the corresponding in vivo experiments. TUNEL analysis demonstrated that obvious apoptosis was present in lymphoma cells treated with CCM@MSNs-ISOIM. Furthermore, the results of the flow cytometry showed cell cycle arrest in the G2/M phase at 48 h, and the Ki-67 analysis in vivo illustrated that CCM@MSNs-ISOIM could inhibit tumor proliferation.

Apoptosis is programmed cell death, which plays an important role in maintaining normal development and cell homeostasis in mammals. Inducing apoptosis in tumor cells is a promising modality in tumor therapies [63]. The smooth progress of the cell cycle is an important condition for cell proliferation; its interference or destruction may prevent cell proliferation and promote cell apoptosis [64]. Numerous studies have confirmed that the active components in traditional Chinese medicine such as alkaloids, flavonoids and saponins may block tumor cells in the G2/M phase [65–67]. In this phase, extracellular DNA damage signals may induce G2/M phase arrest by regulating the activity of the p53 pathway  [68]. The p53-dependent apoptosis pathway is regulated by the p53 protein, which causes mitochondrial release of Cytochrome c, forming apoptosis bodies with Apaf-1 and Pro-Caspase-9, activate Pro-Caspase-9 to form active Caspase-9, and activate effector Caspase-3 through a series of cascade reactions, resulting in tumor cell apoptosis [69]. Here, WB and immunofluorescence were used to explore the mechanism of apoptosis in lymphoma cells induced by CCM@MSNs-ISOIM. The expression of p53, Caspase-9 and Caspase-3 was found to increase significantly, which was more significant than that of the free ISOIM, indicating that the pathway of NHL apoptosis induced by CCM@MSNs-ISOIM may induce tumor cell apoptosis through the p53 pathway. Accordingly, this experiment shows that CCM@MSNs-ISOIM induces NHL to produce high levels of ROS. Similar investigations have shown that high levels of ROS destroys cellular components such as DNA, proteins and lipids, leading to apoptosis [70]. Wang et al. confirmed that Erianin caused a large amount of ROS production and induced G2/M phase arrest in osteosarcoma cells [71]. Recent studies have also shown that the function of p53 is closely related to ROS [72]. ROS not only regulates the activity of p53, but also participates in p53-dependent apoptosis [73]. Therefore, CCM@MSNs-ISOIM may induce the production of high levels of ROS and mediate G2/M block in NHL, which leads to apoptosis.

In addition, classical apoptosis mainly encompasses the mitochondrial and death receptor pathways, of which the mitochondrial pathway is closely related to changes in intracellular mitochondrial transmembrane potential  [74]. CCM@MSNs-ISOIM was found to reduce the intracellular mitochondrial transmembrane potential in NHL, and CCM@MSNs-ISOIM was also observed to significantly increase ROS levels in NHL, further suggesting that CCM@MSNs-ISOIM may induce apoptosis through the intracellular mitochondrial apoptosis pathway. ROS could act on mitochondria, resulting in changes in mitochondrial membrane permeability, decrease in mitochondrial membrane potential and dissociation of cytochrome C from the inner membrane to the intermembrane space [75]. The decrease in mitochondrial membrane potential is a result of the expansion of the mitochondria, which leads to the rupture of the outer membrane, releasing important apoptotic factors into the cytoplasm [76]. Cytochrome C, located in the cytoplasm, initiates the cascade activation of caspase, while apoptosis is initiated by Caspase-3, resulting in the destruction of the entire cell structure, dysfunction and apoptosis [77]. At the same time, high levels of ROS are involved in the destruction of cellular components such as DNA, proteins and lipids, leading to apoptosis.

## Conclusions

In this study, the novel CCM@MSNs-ISOIM embodies a CCM vesicle encapsulated in a MSNs@-ISOIM nanocomplex, which possesses advantages in active targeting of tumor cells, high drug loading rates, high safety, low pH value sensitivity and immune escape, thus being a highly effective and non-toxic treatment modality for use against NHL. This investigation demonstrated that the nanocomplex mediates the blocking of G2/M to activate p53 and modulate the production of high levels of ROS, finally inducing the anti-tumor pathway in mitochondrial apoptosis. In short, this study suggests that CCM@MSNs-ISOIM may provide new treatment options to be used in the treatment of patients suffering from NHL, which may have potentially beneficial clinical outcomes and social benefits.

## Data Availability

All data generated or analyzed during this study are included in this published article.
